# Utility of Keratins as Biomarkers for Human Oral Precancer and Cancer

**DOI:** 10.3390/life12030343

**Published:** 2022-02-25

**Authors:** Milind Vaidya, Crismita Dmello, Saie Mogre

**Affiliations:** 1 Vaidya Laboratory, Advanced Centre for Treatment, Research and Education in Cancer (ACTREC), Tata Memorial Centre (TMC), Kharghar, Navi Mumbai 410210, India; 2 Department of Neurological Surgery, Northwestern Medicine Lou and Jean Malnati Brain Tumor Institute, Robert H. Lurie Comprehensive Cancer Center, Feinberg School of Medicine, Northwestern University, Chicago, IL 60611, USA; 3 Department of Veterinary and Biomedical Sciences, The Pennsylvania State University, State College, PA 16802, USA; sum415@psu.edu

**Keywords:** keratins, oral cancer, oral precancer, biomarker

## Abstract

Human oral cancer is the single largest group of malignancies in the Indian subcontinent and the sixth largest group of malignancies worldwide. Squamous cell carcinomas (SCC) are the most common epithelial malignancy of the oral cavity, constituting over 90% of oral cancers. About 90% of OSCCs arise from pre-existing, potentially malignant lesions. According to WHO, OSCC has a 5-year survival rate of 45–60%. Late diagnosis, recurrence, and regional or lymph nodal metastases could be the main causes of the high mortality rates. Biomarkers may help categorize and predict premalignant lesions as high risk of developing malignancy, local recurrence, and lymph nodal metastasis. However, at present, there is a dearth of such markers, and this is an area of ongoing research. Keratins (K) or cytokeratins are a group of intermediate filament proteins that show paired and differentiation dependent expression. Our laboratory and others have shown consistent alterations in the expression patterns of keratins in both oral precancerous lesions and tumors. The correlation of these changes with clinicopathological parameters has also been demonstrated. Furthermore, the functional significance of aberrant keratins 8/18 expression in the malignant transformation and progression of oral tumors has also been documented. This article reviews the literature that emphasizes the value of keratins as biomarkers for the prognostication of human oral precancers and cancers.

## 1. Classification of Oral Tumors

Oral cancer is a neoplastic disorder in the oral cavity, which includes the following subsites: lip, buccal mucosa, alveolar ridges, retromolar region (gingiva), the floor of the mouth, hard palate, and the anterior/posterior tongue [1]. Oral squamous cell carcinomas (OSCC) are generally classified according to the TNM system by the Union for International Cancer Control [2], where the primary tumor size and extent of tumor growth represents T, regional lymph node metastasis is N, and distant metastasis is M [3,4]. By morphological assessment, tumors are graded as well, moderate, and poorly differentiated [1]. This uniform classifying system is essential for objective evaluation and guiding treatment for oral cancer patients. 

## 2. Development of Oral Precancerous Lesions and Oral Tumors 

Oral squamous cell carcinoma develops through multistep genetic, epigenetic, and metabolic changes resulting from exposure to carcinogens [5]. The precursor lesions, such as oral leukoplakia and submucous fibrosis (SMF), can subsequently progress to cancers; however, only 8–10% of these lesions become malignant [6,7]. The ability to clinically predict malignant transformation is limited, and routine histopathological diagnosis has insufficient prognostic value. The presence of epithelial dysplasia is one of the critical parameters used in the prognostication of leukoplakia. However, the subjective nature of the diagnosis and the observation that both diagnosed and undiagnosed lesions exhibiting leukoplakia may develop to carcinoma or even regress, restricts its use [6]. Therefore, it is necessary to develop supplementary methods to predict the malignant potential of precursor lesions [8]. 

Loco-regional recurrence and regional lymph node metastases are two other contributory factors for poor survival rates of human oral cancers and have proven to be significant hurdles in managing the disease. The presence of lymph nodal metastasis is an important prognostic factor, which can decrease patient survival by 50%. In addition, the accuracy of current clinical and radiological parameters used to predict cervical metastasis is questionable, and there is no consensus on treating the N0 neck [9]. Thus, reliable biomarkers that can predict premalignant lesions at risk of progressing to malignancy or oral tumors that are likely to recur or metastasize, can help prevent the onset of malignancy and delay metastasis through therapeutic intervention.

## 3. Biological Markers 

As an area of ongoing research, we postulate that specific biological markers may help predict patients with a high risk of local recurrence and lymph nodal metastasis. They may also help categorize premalignant lesions as high risk of developing malignancy and improve the management and early detection of oral cancers [10]. In this direction, although the Head and Neck Cancer Genome Anatomy Project (CGAP) has been established, the molecular signals triggering the onset of oral carcinogenesis are still unknown [11]. The current need is to develop reliable, sensitive, and specific markers with the clinical utility to predict high risk oral premalignant lesions. Furthermore, a better understanding of the molecular mechanisms underlying the development of OSCC could add to the treatment and better prognosis of this disease [12]. 

## 4. Keratins

Keratins are the predominant intermediate filament (IF) proteins in epithelial cells. They are expressed in pairs and are differentiation-dependent and tissue-specific [13]. Keratins account for 80% of the total protein content of stratified epithelia [14]. The first keratin protein nomenclature was published as a comprehensive keratin catalog by Moll et al. in 1982 [15]. At the time, this catalog included 19 members of the keratins family, which were further classified as type I and II keratins. Keratins 9 to 19 are the type I IF proteins. These are acidic (PI 4.5–5.5), with low molecular weight (40–56.6 kDa). Keratins 1 to 8 are the type II IF proteins. These are basic to neutral (PI 5.5–7.8) and have higher molecular weights (53–68 kDa). Additional keratins were subsequently identified in humans and in other species, and the keratin catalog has been updated several times [16,17]. Since the completion of the human genome sequence, an updated nomenclature for mammalian keratin genes and proteins is now available [18]. It includes 28 type I (20 epithelial and 8 hair) keratins and 26 type II (20 epithelial and 6 hair) keratins. 

## 5. Keratin Expression in Normal Human Oral Cavities

**Figure 1:**
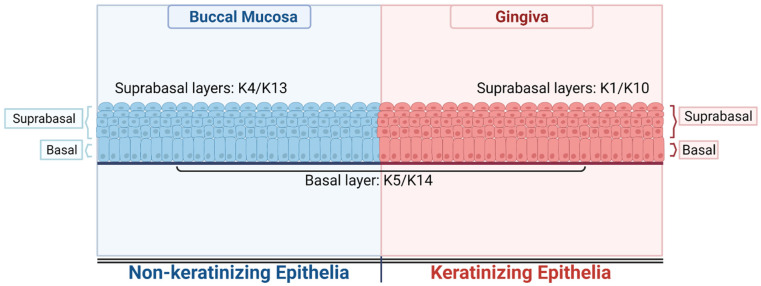
**Differentiation specific expression of keratins in oral cavity.** The illustration shows expression of keratins in the suprabasal and basal layers of nonkeratinizing epithelia–Buccal mucosa and in the suprabasal and basal layers of keratinizing epithelia–gingiva. (Created with BioRender-Available online: https://biorender.com/, accessed on 4 February 2022).

The human oral cavity is one of the best examples to illustrate the differentiation-dependent expression of keratin in a stratified epithelia. Various sites in the human oral cavity show distinct levels of differentiation and keratinization, and a corresponding variation in the keratin expression. All the sites in the human oral cavity express the keratin pair K5 and K14 in the basal layer; however, expression of other keratin pairs, such as K1 and K10 or K4 and K13, in the suprabasal layers follows [19]. For example, as shown in Figure 1, buccal mucosa, a nonkeratinizing epithelium, expresses a keratin pair of K4 and K13 in the suprabasal layers, whereas the basal layer expresses a keratin pair of K5 and K14. Gingiva, a keratinizing epithelium, expresses K1 and K10 in the suprabasal layers, while the basal layer shows the expression of keratin pair K5 and K14 [20]. This differential expression of differentiation specific keratins in keratinizing and nonkeratinizing epithelia has functional implication. For example, K1/10 have high molecular weight and form dense bundles that provide mechanical strength to the epidermal tissue and inhibits the proliferation of the keratinocytes. Hence, mutations or loss in K1/10 are associated with skin blistering and hyperproliferation [21,22]. On the other hand, the nonkeratinizing epithelia specific keratin pair K4/13 has a comparatively lower molecular weight than K1/10 and functions to provide strength to the mucosal stratified squamous epithelia. Mutations in these keratins are manifested as white plaques on the buccal mucosa [21]. Keratins are being widely used in clinics as tumor markers to aid in the histodiagnosis and management of certain cancers [23,24,25]. These proteins hold a unique position due to their abundance and tissue-specific and differentiation-dependent expression in the epithelial cells. [15,19,26,27,28,29,30,31,32,33,34].

## 6. Detection of Keratins

### 6.1. Transcription

Keratin typing or fingerprinting [35] is widely used to identify simple and stratified epithelia, characterize normal and transformed epithelia, mark invasive and noninvasive tumor margins, and predict patient survival. In another study, Bosch et al. [31] have shown changes in the keratin expression pattern and their corresponding mRNAs in pathologically altered oral gingiva. They noted an increase in the number of cells producing keratins 4 and 13 coupled with a reduction in the number of cells producing keratins 1, 10, and 11. Moreover, an increase in the expression of keratin 19 was seen in the basal as well as suprabasal layers. These observations indicated that keratin expression might be altered during and after the malignant transformation of the oral mucosa. In another study, Yonemochi et al. compared the profiles for K13 and K16, which are not expressed in normal epithelia. They reported that these are enhanced in carcinoma in situ (CIS) using in situ hybridization and RT-PCR in formalin fixed paraffin sections of CIS. Their results suggest that the loss of K13 can either be attributed to its gene repression or an unknown post-translational event [36]. Su et al. reported a lower expression of mRNAs for K7, K8, and K18 in the basal layer of normal oral epithelia. Weak mRNA and protein expression for keratins 8 and 18 were observed in the suprabasal layers of dysplastic oral leukoplakia tissues that was reversed in OSCC, suggesting that the transcriptional or repression block on keratin expression is removed under these conditions [37]. 

### 6.2. Proteomics

The initial efforts of researchers focused on cataloging the keratin expression profiles in both premalignant and malignant lesions and comparing their expression to normal tissues. Several studies have shown and cataloged specific keratin subtypes as downregulated or upregulated in oral premalignant lesions and oral squamous cell carcinoma (OSCC) using biochemical methods such as immunoblotting and 2-D gel electrophoresis [38,39,40]. Ermich et al. [38] reported altered keratin expression in the leukoplakia and SCC of oral mucosa using SDS-PAGE. Although the pattern of basic keratins remained unchanged compared to normal tissue, abnormal expression of K18 and K19 was observed in some samples. However, these alterations did not correlate to the degree of dysplasia. In 1989, we showed differentiation-dependent changes in keratin expression pattern in SCC of buccal mucosa using SDS-PAGE and 2-D gel electrophoresis [39]. Our results showed aberrant expression of keratins 17, 18, and 19 in moderately differentiated SCC, while aberrant expression of K1 and K16 was seen predominantly in well differentiated SCC. Both types of tumors consistently showed the non-expression of keratin pair K5 and K13. Importantly, some of the SCCs also demonstrated isoelectric point variants. In another study, we demonstrated altered keratin expression in the SCC of tongue and alveolar mucosa (AM). Our results showed that keratins such as K4, K5, and K14 were not expressed in SCC at both these sites, while other keratins, such as K1 and K18, were aberrantly expressed. Thus, non-expression of basic keratin K5 in the epithelia of the oral lining and aberrant expression of simple epithelial keratins seem to be the major events in the malignant transformation of the oral epithelium [41]. We have also reported the non-expression of keratin pair K5 and K14 in most leukoplakia and oral submucous fibrosis (OSMF) samples [40] and an aberrant expression of simple epithelia specific K8 in a few leukoplakia and OSMF samples. Overall, our studies have highlighted the non-expression of stratified epithelia specific keratin pair of 5/14 as tobacco related.

Some researchers have used large-scale proteomic analysis to characterize oral tissues as normal, precancerous, and SCCs. In one such study, Gires et al. screened head and neck carcinoma patients with the proteomics based AMIDA technology. It yielded a set of tumor associated antigens, including keratin 8, which positively correlated to the malignancies of the head and neck. Interestingly, de novo expression of K8 also correlated to the dysplastic areas of oral leukoplakic lesions, while hyperplastic leukoplakia remained K8 negative. Thus, K8 was proposed as an attractive marker for the differential diagnosis of leukoplakia and head and neck carcinomas [42]. To explore the presence of differentially expressed proteins in OSCC and enable discrimination between the normal and tumor mucosa, 12 paired normal and tumor samples were analyzed using 2-D gel electrophoresis and MALDI-TOF. Some keratins, such as 5, 6, 13, 14, 16, 17, and 19, were differentially expressed [43]. Chiang et al. analyzed differential proteomes between oral cancer cells and the areca nut extract treated cells using isobaric mass tag (iTRAQ) labeling and LC-MS mass spectrometry. K17 was identified as one of the differentially expressed proteins. A murine carcinogen treated model also showed an elevated expression of K17 in hyperplastic and carcinoma tissues. Furthermore, K17 knockdown significantly suppressed areca nut extract induced cell migration and invasion and modulated the EMT process. The authors conclude that K17 contributes to areca nut-induced oral malignancy [44].

### 6.3. Immunohistochemistry

With the advent of monospecific monoclonal antibodies against several keratins, immunohistochemistry became the method of choice to detect changes in keratin expression during and after the malignant transformation of the oral cavity. IHC allowed visualizing the localization of keratins, which was useful in multilayered epithelial tissue such as the oral mucosa, which expresses different subsets of keratins. Keratin IHC analysis using monoclonal antibodies was successfully demonstrated by Ogden et al. using fresh tissue sections to detect subtle changes in epithelial differentiation that were not visible by H&E staining [45]. Similar changes were also observed in clinically normal epithelium from oral cancer patients, primarily in the expression of keratins K8 and K7 in the basal cells of tumor biopsies. Moreover, a trend towards reducing the complexity of keratin differentiation was also noted. Examining changes in keratin expression by IHC is a valuable adjunct to H&E staining, given its prognostic and diagnostic significance. 

## 7. Aberrant Keratin Expression in Oral Precancerous Lesions 

Lindberg et al. have shown the suprabasal expression of K19 in the dysplastic keratinizing and nonkeratinizing tissues, and the SCC of the oral mucosa [29], which may be linked to the retention of stem-cell characteristics typical of keratinocytes that are uncommitted to terminal differentiation. Thus, K19 presents itself as a valuable tool in oral histopathologic diagnosis. Bloor et al. [46] investigated the differentiation-specific keratins (K4, K13, K1, and K10) in oral epithelial dysplasia and squamous cell carcinoma (SCC). In severe dysplasia and poorly differentiated SCC, keratin gene expression reflected the gross changes in epithelial differentiation and maturation. A lower expression of K14 mRNA was seen in OSCC tissues compared to normal and white patch tissues with dysplasia, indicating that K14 downregulation is a late event in human oral carcinogenesis [47]. Along the same line, non-expression of keratin pair of 5/14 in the majority of leukoplakia and OSMF samples was reported [40]. Aberrant expression of simple epithelia-specific K8 in a few leukoplakia and OSMF samples was also demonstrated. The authors concluded that non-expression of the stratified epithelia specific keratin pair of 5/14 was a tobacco related change. Ranganathan et al. studied the keratin profile in OSMF tissues to ascertain if keratins can serve as a marker for malignant transformation [48]. They found a significant difference in the keratin staining pattern between normal, OSMF, and cancer. Significant changes in OSMF included increased intensity of staining for pan keratin and high molecular weight keratin, aberrant expression of K8, and decreased expression of K5 and K14. These results indicate their potential as the surrogate markers of malignant transformation.

In another study, the investigators aimed to understand if the loss of expression of K4, K13, and cornulin can predict malignant transformation from oral leukoplakia; however, they reported no significant correlation between the keratins and malignant transformation, in complete contrast to several other studies. A likely explanation for the discrepancy could be that the aberrant differentiation state of hyperkeratotic leukoplakia lesions had already decreased the keratin expression, thus lowering its value to predict its malignant potential [49]. However, the aberrant expression of keratin 17 and non-expression of K13 were the two changes observed in the severe dysplasia and SCC tissues of the human oral mucosa. Normal mild dysplasia showed the expression of K13, while K17 was not detected in these tissues [50]. In a subset of the most severe OSMF cases (14%) proposed to be at most risk of undergoing malignant transformation, K17 expression was lost in the basal layer. In addition, the increase in K1 and K10 in the suprabasal layers, induction of K6 in the basal layer, and complete loss of K19 in the epithelium in SMF samples in comparison with normal tissues were some of the salient features of the study by Lalli et al. [51]. A study by Bloor et al. showed the presence of both sets of differentiation keratins (K4/13 and K1/10) in mild dysplasia and in tumor islands of well and moderately differentiated SCC. In moderate lesions, the expression of K4 and K13 was reduced in the presence of enhanced K1 and K10 synthesis, whereas K1/K10 mRNA and protein expression was lost in severe dysplasia and poorly differentiated SCC of the oral cavity [11]. 

## 8. Aberrant Expression of Keratins in OSCC

Sakamoto et al. analyzed the expression of all the keratins in OSCC samples by cDNA microarray and the immunohistochemistry of OSSC and oral epithelial dysplasia (OED), to identify relevant keratin subtypes associated with the pathogenesis of oral epithelial neoplasms. They observed consistent non-expression of keratin pair K4/K13 in SCC and OED. Additionally, in vitro transfection of various keratins in oral epithelial cell lines showed changes in cell shape and movement, leading to the conclusion that these changes arise due to the dysregulation of cell differentiation and can be used to prognosticate oral cancers [52]. Furthermore, Farrar et al. proved that lower AE1/AE3 and K14 expression was a late event in oral carcinogenesis, particularly in poorly differentiated SCC [53]; cell lines derived from the SCC of the human tongue revealed that the downregulation or absence of keratins 13, 14 and 16 is associated with an invasive and metastatic phenotype of the cells [54]. In addition, Wei et al. have shown increased expression of K17 in OSCC-derived cell lines and tissues, compared to their normal counterparts [55]. Consistent with these studies, we have demonstrated that vimentin regulates differentiation switching via the modulation of K5/14 transcript levels, perhaps via ΔNP63 signaling. The expression of K5/14 and vimentin correlated with a poor prognosis of oral cancer patients [56,57].

A study by Schulz et al. has reported marked aberrations from normal keratin expression characterized by the appearance of K8, K18, and K19, proteolytic modifications of keratins, and partial loss of site specific keratins in leukoplakia and mucosal squamous cell carcinoma [58]. Furthermore, Ogden et al. showed that simple epithelial keratins expression (K8, K18, and K19) was not confined to poorly-differentiated tumors [59] and can also be relevant to oral cancer prognosis. For example, increased K19 expression was detected in hyperplastic, dysplastic, and malignant lesions, whereas K8 expression increased only in dysplastic and malignant lesions [60]. Zhong et al. reported a correlation between increased K19 mRNA and protein expression in OSCC tissue with the pathological differentiation grade. In their study, K19 expression in surrounding uninvolved tissue suggested a higher tumor recurrence and a lower survival rate [61]. Cintorino et al. have demonstrated the presence of K19 in both the basal and suprabasal layers of severely dysplastic nonkeratinizing oral tissues and poorly differentiated OSCC, while K1 expression was limited to the suprabasal layers [62]. Increased keratin 19 expression was detected in EBV infected oral SCCs [63]. In addition, K8 expression was detected in the early stages of the disease, that is, the dysplastic oral leukoplakia, but in not the normal or hyperplastic epithelium of the head and neck area, including the normal mucosa, hyperplastic and dysplastic leukoplakia, carcinomas, and lymph node metastases [64], as well as in carcinomas originating from other regions except those of the larynx and the tongue. Interestingly, K8^high^ was a characteristic of invading cells detached from the primary tumors, thus proving K8 as an excellent marker for OSCC and metastasis.

Crowe et al. also observed abundant K19 expression in normal and dysplastic oral tissues, which was patchy or absent in invasive OSCC. They further showed K19 downregulation in seven invasive and metastatic cell lines, while forced expression of K19 in highly metastatic cells lowered their invasive potential. While these findings vary from some of the other studies [65], together, K19 and K8 could be markers of the sequential premalignant changes in head and neck carcinogenesis [60].

## 9. Correlation of Keratin Expression with Clinicopathological Parameters 

A study conducted by Depondt et al. on 26 OSCC samples associated positive or negative expression of specific keratins with a better prognosis. They reported an association between K10 and K19 expression with smaller tumor size and a negative association between K18 expression to lymph node metastasis [66]. The immunohistochemical analysis of frozen sections of 120 human mucosal squamous cell carcinomas from various topological sites showed a higher expression of keratins 8/18 in more than 50% of the tumors. In addition, the analysis also revealed a characteristic intermediate filament phenotype distinct from the differentiation specific pattern in the invasive carcinoma cells interacting with the stromal microenvironment. These changes might contribute to the invasive, migratory, and proliferative phenotype of the cells [67].

A large-scale study by Fillies et al. analyzed the expression of keratins 5, 6, 8, 18, 1, 10, 14, and 19 by tissue microarrays of oral leukoplakia and OSCC with known clinicopathological parameters. The tissue microarray analysis investigated 192 patients with OSCC, 117 patients with oral leukoplakia without dysplasia, and 23 oral leukoplakia with dysplasia (or squamous intraepithelial neoplasia) of the oral cavity. It showed that the aberrant expression of K1, K8/18, and K19 correlated with the degree of dysplasia and the acquisition of growth properties. They also concluded that the expression of K8/K18 and K19 in transformed oral lesions could be an early feature in the pathogenesis of invasive OSCC [12]. In another study, the authors investigated 308 patients with histologically proven and surgically treated SCC of the oral cavity for the immunohistochemical expression of keratins 5/6, 8/18, 1/10, 14, 19, and vimentin using the tissue microarray. The analysis demonstrated that the expression of K8/18 and K19 significantly correlated to a poor clinical prognosis. These findings were corroborated in a multivariate analysis of K8/18 expression in primary nodal negative SCCs. Overall, the investigators concluded that K8/18 expression in oral SCC is an independent prognostic marker that can indicate a lower overall and progression free survival [68]. Our laboratory has also shown the prognostic significance of vimentin in oral cancer and premalignancy. Therefore, using a combination of keratins and vimentin expression can add substantial value to better predict a prognosis of oral precancer and cancer [69,70,71].

Kitamura et al. analyzed the expression patterns of K17 and K13 in OSCC and leucoplakia using the immunohistochemistries of 105 patients with OSCC and 108 patients with leucoplakia and reported an association between K17 and the differentiation and malignancy of OSCC. In addition to IHC, real time RT-PCR from five OSCC cell lines showed the overexpression of K17 mRNA, suggesting the potential of K13/K17 expression as a marker for malignant transformation [72]. Sawant et al. conducted a study to discover a potential correlation between the clinicopathological parameters of patients with potentially malignant lesions and the immunohistochemical expression and localization of keratins 1, 5, 8, and 18. The analysis conclusively proved that a non-expression of K5 and aberrant expression of K1, K8, and K18 independently and significantly correlated with the clinicopathological progressive grade and some of the clinicopathological factors of oral premalignant disorders [73]. These findings suggest that the alterations in the expression pattern of keratins could serve as a surrogate marker for diagnosing potentially malignant oral disorders and may also have prognostic value in patients with oral cancer.

Furthermore, RT-PCR and IHC analysis targeting keratins 5, 14, and 17 on the sentinel lymph nodes (SLN) of oral and oropharyngeal carcinomas revealed higher mRNA expression than negative SLNs. K17 was predicted as an accurate marker for diagnosing micrometastases of a size greater than 450 Am. Thus, quantitative RT-PCR for SLN staging in cN0 patients with oral and oropharyngeal squamous cell carcinoma is promising [74]. 

To summarize, consistent quantitative and qualitative alterations in both the mRNA and protein expression of keratins have been shown in oral precancerous lesions and SCC. Two major alterations are (1) changes in differentiation related keratins and (2) aberrant expression of simple epithelial keratins 8, 18, and 19. Various studies have established correlations between clinicopathological parameters and the aberrant expression of simple epithelial keratins 8/18, thus, the keratins pair K8/18 shows the highest potential as a biomarker.

## 10. Functional Significance of Aberrant Keratin 8/18 Expression in Oral Precancer and Cancer

K8/18 is the first keratin pair expressed during embryogenesis [75], and the pair is unique in many ways. All type I keratin genes are located on chromosome 17, while all type II keratin genes are situated on chromosome 12. Although K8/18 belong to the type II and type I subfamilies, respectively, both are located on chromosome 12 [76]. The expression of K18 is regulated by AP1 and ETS families of transcription factors, which also regulate the expression of the Ras oncogenes [76]. Forced expression of K8/18 and vimentin in mouse fibroblasts increased resistance to various chemotherapeutic drugs [77]. K8/18- and vimentin-expressing melanomas are more invasive than those expressing only vimentin [78]. This is consistent with the in vitro behavior in human melanoma cells that resulted in increased migratory and invasive activity upon the overexpression of K8/18 [79]. Mouse fibroblasts expressing K8 and K18 also exhibited a higher migratory and invasive ability [80]. Keratin 8 transfection to FBM cells, a cell line derived from the human fetal buccal mucosa that expresses K8 and K18 in a ratio of 3:1 [81], showed the proper formation of keratin 8/18 filaments, a reduction in doubling time, cell clusters with projections, and anchorage independent growth in soft agar colony assay. Xenografts using K8 transfected FBM cells also produced tumors in nude mice with lung metastasis. In addition, transgenic mice expressing human K8 in the epidermis showed a dramatic increase in the progression of papilloma towards malignancy [82]. Another report showed the modulation of integrin and FAK mediated cell migration by K8 and K18 through protein kinase C (PKC) in hepatocytes [83]. Combined, these suggest that aberrant expression of K8 and K18 contributes to the neoplastic progression in various tissues, including stratified epithelial cells. 

Keratins can interact with both the desmosomes at the cell–cell junctions via desmoplakin and the hemidesmosome at the cell–ECM junctions via the α6β4 integrin that further links to plectin and BP230 [84,85]. In addition, the type III intermediate protein vimentin can also interact with α6β4 integrin, perhaps via plectin in the OSCC derived cell line [86]. Our laboratory has demonstrated that a knockdown of K8 in the OSCC derived cell line AW13516 leads to a substantial reduction in tumorigenicity, cell motility, cell invasion, fascin, and α6β4 integrin expression and signaling. Taken together, our results proved a role of K8 and K18 to promote malignant transformation and play an critical role in invasion and metastasis, possibly by modulating signaling through β4 integrin [87].

## 11. Conclusions

The value of keratins as diagnostic markers in epithelial malignancies has already been established. Alterations in keratin expression and its significance in precancerous lesions and tumors have been conclusively illustrated using biochemical, immunohistochemical, and other molecular biology techniques, and the expression patterns correlate with many clinicopathological parameters. The literature reviewed in this article underlines their importance as prognostic markers for both oral precancer and cancer.

## Data Availability

Not applicable.

## References

[1] Hillbertz N.S., Hirsch J.A.N.M., Jalouli J., Jalouli M.M., Sand L. (2012). Viral and Molecular Aspects of Oral Cancer. Anticancer Res..

[2] Sobin L.H., Gospodarowicz M.K., Wittekind C., Sobin L.H., Gospodarowicz M.K., Wittekind C., Internation Union Against Cancer (2009). TNM Classification of Malignant Tumours.

[3] Leemans C.R., Braakhuis B.J.M., Brakenhoff R.H. (2011). The molecular biology of head and neck cancer. Nat. Rev. Cancer.

[4] Massano J., Regateiro F.S., Januário G., Ferreira A. (2006). Oral squamous cell carcinoma: Review of prognostic and predictive factors. Oral Surg. Oral Med. Oral Pathol. Oral Radiol. Endodontol..

[5] Lippman S.M., Hong W.K. (2001). Molecular Markers of the Risk of Oral Cancer. N. Engl. J. Med..

[6] Reibel J. (2003). Prognosis of Oral Pre-malignant Lesions: Significance of Clinical, Histopathological, and Molecular Biological Characteristics. Crit. Rev. Oral Biol. Med..

[7] Gupta P., Bhonsle R., Murti P., Daftary D., Mehta F., Pindborg J. (1989). An Epidemiologic Assessment of Cancer Risk in Oral Precancerous Lesions in India With Special Reference to Nodular Leukoplakia. Cancer.

[8] Allison P., Locker D., Feine J.S. (1998). The role of diagnostic delays in the prognosis of oral cancer: A review of the literature. Oral Oncol..

[9] Cerezo L., Millan I., Torre A., Aragon G., Otero J. (1992). Prognostic factors for survival and tumor control in cervical lymph node metastases from head and neck cancer. A multivariate study of 492 cases. Cancer.

[10] Jemal A., Tiwari R.C., Murray T., Ghafoor A., Samuels A., Ward E., Feuer E.J., Thun M.J. (2004). Cancer statistics, 2004. CA Cancer J. Clin..

[11] Bloor B., Seddon S., Morgan P. (2001). Gene expression of differentiation-specific keratins in oral epithelial dysplasia and squamous cell carcinoma. Oral Oncol..

[12] Fillies T., Jogschies M., Kleinheinz J., Brandt B., Joos U., Buerger H. (2007). Cytokeratin alteration in oral leukoplakia and oral squamous cell carcinoma. Oncol. Rep..

[13] Coulombe P.A., Omary M.B. (2002). ‘Hard’ and ‘soft’ principles defining the structure, function and regulation of keratin intermediate filaments. Curr. Opin. Cell Biol..

[14] Pekny M., Lane E. (2007). Intermediate filaments and stress. Exp. Cell Res..

[15] Moll R., Franke W.W., Schiller D.L., Geiger B., Krepler R. (1982). The catalog of human cytokeratins: Patterns of expression in normal epithelia, tumors and cultured cells. Cell.

[16] Takahashi K., Paladini R.D., Coulombe P.A. (1995). Cloning and Characterization of Multiple Human Genes and cDNAs Encoding Highly Related Type II Keratin 6 Isoforms *. J. Biol. Chem..

[17] Moll R., Schiller D.L., Franke W.W. (1990). Identification of protein IT of the intestinal cytoskeleton as a novel type I cytokeratin with unusual properties and expression patterns. J. Cell Biol..

[18] Schweizer J., Bowden P.E., Coulombe P.A., Langbein L., Lane E.B., Magin T.M., Maltais L., Omary M.B., Parry D.A.D., Rogers M.A. (2006). New consensus nomenclature for mammalian keratins. J. Cell Biol..

[19] Morgan P.R., Shirlaw P.J., Johnson N.W., Leigh I.M., Lane E.B. (1987). Potential applications of anti-keratin antibodies in oral diagnosis. J. Oral Pathol..

[20] Moll R., Divo M., Langbein L. (2008). The human keratins: Biology and pathology. Histochem. Cell Biol..

[21] Lane E.B., McLean W.H. (2004). Keratins and skin disorders. J. Pathol..

[22] Omary M.B., Coulombe P.A., McLean W.H. (2004). Intermediate filament proteins and their associated diseases. N. Engl. J. Med..

[23] Moll R., Krepler R., Franke W.W. (1983). Complex cytokeratin polypeptide patterns observed in certain human carcinomas. Differ. Res. Biol. Divers..

[24] Moll R. (1987). Diversity of cytokeratins in carcinomas. Acta Histochem..

[25] Osborn M., van Lessen G., Weber K., Kloppel G., Altmannsberger M. (1986). Differential diagnosis of gastrointestinal carcinomas by using monoclonal antibodies specific for individual keratin polypeptides. Lab. Investig. A J. Tech. Methods Pathol..

[26] Lazarides E. (1980). Intermediate filaments as mechanical integrators of cellular space. Nature.

[27] Osborn M., Weber K. (1982). Intermediate filaments: Cell-type-specific markers in differentiation and pathology. Cell.

[28] Blobel G.A., Moll R., Franke W.W., Kayser K.W., Gould V.E. (1985). The intermediate filament cytoskeleton of malignant mesotheliomas and its diagnostic significance. Am. J. Pathol..

[29] Lindberg K., Rheinwald J.G. (1989). Suprabasal 40 kd keratin (K19) expression as an immunohistologic marker of premalignancy in oral epithelium. Am. J. Pathol..

[30] Vigneshwaran N., Peters K.-P., Hornstein O.P., Haneke E. (1989). Comparison of cytokeratin, filaggrin and involucrin profiles in oral leukoplakias and squamous carcinomas. J. Oral Pathol. Med..

[31] Bosch F.X., Ouhayoun J.P., Bader B.L., Collin C., Grund C., Lee I., Franke W.W. (1989). Extensive changes in cytokeratin expression patterns in pathologically affected human gingiva. Virchows Arch..

[32] Heyden A., Huitfeldt H.S., Koppang H.S., Thrane P.S., Bryne M., Brandtzaeg P. (1992). Cytokeratins as epithelial differentiation markers in premalignant and malignant oral lesions. J. Oral Pathol. Med..

[33] Coulombe P.A. (1993). The cellular and molecular biology of keratins: Beginning a new era. Curr. Opin. Cell Biol..

[34] Fuchs E., Weber K. (1994). Intermediate filaments: Structure, dynamics, function, and disease. Annu. Rev. Biochem..

[35] McDonald L.A., Walker D.M., Gibbins J.R. (1998). Cervical lymph node involvement in head and neck cancer detectable as expression of a spliced transcript of type II keratin K5. Oral Oncol..

[36] Ida-Yonemochi H., Maruyama S., Kobayashi T., Yamazaki M., Cheng J., Saku T. (2012). Loss of keratin 13 in oral carcinoma in situ: A comparative study of protein and gene expression levels using paraffin sections. Mod. Pathol..

[37] Su L., Morgan P.R., Lane E.B. (1994). Protein and mRNA expression of simple epithelial keratins in normal, dysplastic, and malignant oral epithelia. Am. J. Pathol..

[38] Ermich T., Schulz J., Raabe G., Schumann D. (1989). Pattern of oral cytokeratins. III. SDS-electrophoretic analysis and immunoblotting of cytokeratins in leukoplakias and squamous cell carcinomas of the oral mucosa. Biomed. Biochim. Acta.

[39] Vaidya M.M., Borges A.M., Pradhan S.A., Rajpal R.M., Bhisey A.N. (1989). Altered keratin expression in buccal mucosal squamous cell carcinoma. J. Oral Pathol. Med. Off. Publ. Int. Assoc. Oral Pathol. Am. Acad. Oral Pathol..

[40] Vaidya M.M., Sawant S.S., Borges A.M., Ogale S.B., Bhisey A.N. (1998). Cytokeratin expression in precancerous lesions of the human oral cavity. Oral Oncol..

[41] Vaidya M.M., Borges A.M., Pradhan S.A., Bhisey A.N. (1996). Cytokeratin expression in squamous cell carcinomas of the tongue and alveolar mucosa. Eur. J. Cancer B Oral Oncol..

[42] Gires O., Mack B., Rauch J., Matthias C. (2006). CK8 correlates with malignancy in leukoplakia and carcinomas of the head and neck. Biochem. Biophys. Res. Commun..

[43] Roman E., Lunde M.L.S., Miron T., Warnakulasauriya S., Johannessen A.C., Vasstrand E.N., Ibrahim S.O. (2013). Analysis of Protein Expression Profile of Oral Squamous Cell Carcinoma by MALDI-TOF-MS. Anticancer Res..

[44] Chiang C.-H., Wu C.-C., Lee L.-Y., Li Y.-C., Liu H.-P., Hsu C.-W., Lu Y.-C., Chang J.T., Cheng A.-J. (2016). Proteomics Analysis Reveals Involvement of Krt17 in Areca Nut-Induced Oral Carcinogenesis. J. Proteome Res..

[45] Ogden G.R., Lane E.B., Hopwood D.V., Chisholm D.M. (1993). Evidence for field change in oral cancer based on cytokeratin expression. Br. J. Cancer.

[46] Bloor B.K., Seddon S.V., Morgan P.R. (2000). Gene expression of differentiation-specific keratins (K4, K13, K1 and K10) in oral non-dysplastic keratoses and lichen planus. J. Oral Pathol. Med. Off. Publ. Int. Assoc. Oral Pathol. Am. Acad. Oral Pathol..

[47] Marley J.J., Robinson P.A., Hume W.J. (1994). Expression of human cytokeratin 14 in normal, premalignant and malignant oral tissue following isolation by plaque differential hybridisation. Eur. J. Cancer Part B Oral Oncol..

[48] Ranganathan K., Kavitha R., Sawant S.S., Vaidya M.M. (2006). Cytokeratin expression in oral submucous fibrosis—An immunohistochemical study. J. Oral Pathol. Med..

[49] Schaaij-Visser T.B.M., Bremmer J.F., Braakhuis B.J.M., Heck A.J.R., Slijper M., van der Waal I., Brakenhoff R.H. (2010). Evaluation of cornulin, keratin 4, keratin 13 expression and grade of dysplasia for predicting malignant progression of oral leukoplakia. Oral Oncol..

[50] Mikami T., Cheng J., Maruyama S., Kobayashi T., Funayama A., Yamazaki M., Adeola H.A., Wu L., Shingaki S., Saito C. (2011). Emergence of keratin 17 vs. loss of keratin 13: Their reciprocal immunohistochemical profiles in oral carcinoma in situ. Oral Oncol..

[51] Lalli A., Tilakaratne W., Ariyawardana A., Fitchett C., Leigh I., Hagi-Pavli E., Cruchley A., Kenneth E., Teh M.-T., Fortune F. (2008). An altered keratinocyte phenotype in oral submucous fibrosis: Correlation of keratin K17 expression with disease severity. J. Oral Pathol. Med. Off. Publ. Int. Assoc. Oral Pathol. Am. Acad. Oral Pathol..

[52] Sakamoto K., Aragaki T., Morita K., Kawachi H., Kayamori K., Nakanishi S., Omura K., Miki Y., Okada N., Katsube K. (2011). Down-regulation of keratin 4 and keratin 13 expression in oral squamous cell carcinoma and epithelial dysplasia: A clue for histopathogenesis. Histopathology.

[53] Farrar M., Sandison A., Peston D., Gailani M. (2004). Immunocytochemical analysis of AE1/AE3, CK 14, Ki-67 and p53 expression in benign, premalignant and malignant oral tissue to establish putative markers for progression of oral carcinoma. Br. J. Biomed. Sci..

[54] Morifuji M., Taniguchi S., Sakai H., Nakabeppu Y., Ohishi M. (2000). Differential expression of cytokeratin after orthotopic implantation of newly established human tongue cancer cell lines of defined metastatic ability. Am. J. Pathol..

[55] Wei K.J., Zhang L., Yang X., Zhong L.P., Zhou X.J., Pan H.Y., Li J., Chen W.-R., Zhang Z.Y. (2009). Overexpression of cytokeratin 17 protein in oral squamous cell carcinoma in vitro and in vivo. Oral Dis..

[56] Dmello C., Sawant S., Alam H., Gangadaran P., Mogre S., Tiwari R., D’Souza Z., Narkar M., Thorat R., Patil K.J.P.O. (2017). Vimentin regulates differentiation switch via modulation of keratin 14 levels and their expression together correlates with poor prognosis in oral cancer patients. PLoS ONE.

[57] Mogre S., Makani V., Pradhan S., Devre P., More S., Vaidya M., Dmello C. (2022). Biomarker Potential of Vimentin in Oral Cancers. Life.

[58] Schulz J., Ermich T., Kasper M., Raabe G., Schumann D. (1992). Cytokeratin pattern of clinically intact and pathologically changed oral mucosa. Int. J. Oral Maxillofac. Surg..

[59] Ogden G.R., Chisholm D.M., Adi M., Lane E.B. (1993). Cytokeratin expression in oral cancer and its relationship to tumor differentiation. J. Oral Pathol. Med. Off. Publ. Int. Assoc. Oral Pathol. Am. Acad. Oral Pathol..

[60] Xu X.C., Lee J.S., Lippman S.M., Ro J.Y., Hong W.K., Lotan R. (1995). Increased expression of cytokeratins CK8 and CK19 is associated with head and neck carcinogenesis. Cancer Epidemiol. Biomark. Prev..

[61] Zhong L.-P., Chen W.-T., Zhang C.-P., Zhang Z.-Y. (2007). Increased CK19 expression correlated with pathologic differentiation grade and prognosis in oral squamous cell carcinoma patients. Oral Surg. Oral Med. Oral Pathol. Oral Radiol. Endod..

[62] Cintorino M., Petracca R., Vindigni C., Tripodi S.A., Leoncini P. (1990). Topography-related expression of individual cytokeratins in normal and pathological (non-neoplastic and neoplastic) human oral mucosa. Virchows Arch. A.

[63] Jiang W.G., Watkins G., Douglas-Jones A., Holmgren L., Mansel R.E. (2006). Angiomotin and angiomotin like proteins, their expression and correlation with angiogenesis and clinical outcome in human breast cancer. BMC Cancer.

[64] Matthias C., Mack B., Berghaus A., Gires O. (2008). Keratin 8 expression in head and neck epithelia. BMC Cancer.

[65] Crowe D.L., Milo G.E., Shuler C.E. (1999). Keratin 19 Downregulation by Oral Squamous Cell Carcinoma Lines Increases Invasive Potential. J. Dent. Res..

[66] Depondt J., Shabana A.H., Sawaf H., Gehanno P., Forest N. (1999). Cytokeratin alterations as diagnostic and prognostic markers of oral and pharyngeal carcinomas. A prospective study. Eur. J. Oral Sci..

[67] Schaafsma H.E., Van Der Velden L.A., Manni J.J., Peters H., Link M., Rutter D.J., Ramaekers F.C. (1993). Increased expression of cytokeratins 8, 18 and vimentin in the invasion front of mucosal squamous cell carcinoma. J. Pathol..

[68] Fillies T., Werkmeister R., Packeisen J., Brandt B., Morin P., Weingart D., Joos U., Buerger H. (2006). Cytokeratin 8/18 expression indicates a poor prognosis in squamous cell carcinomas of the oral cavity. BMC Cancer.

[69] Dmello C., Sawant S., Chaudhari P.R., Dongre H., Ahire C., D’Souza Z.C., Charles S.E., Rane P., Costea D.E., Chaukar D.J.E. (2018). Aberrant expression of vimentin predisposes oral premalignant lesion derived cells towards transformation. Exp. Mol. Pathol..

[70] Sawant S., Vaidya M., Chaukar D., Alam H., Dmello C., Gangadaran P., Kannan S., Kane S., Dange P., Dey N. (2014). Clinical significance of aberrant vimentin expression in oral premalignant lesions and carcinomas. Oral Dis..

[71] Dmello C., Srivastava S.S., Tiwari R., Chaudhari P.R., Sawant S., Vaidya M.M. (2019). Multifaceted role of keratins in epithelial cell differentiation and transformation. J. Biosci..

[72] Kitamura R., Toyoshima T., Tanaka H., Kawano S., Kiyosue T., Matsubara R., Goto Y., Hirano M., Oobu K., Nakamura S. (2012). Association of cytokeratin 17 expression with differentiation in oral squamous cell carcinoma. J. Cancer Res. Clin. Oncol..

[73] Sawant S., Vaidya M., Chaukar D., Gangadaran P., Singh A., Rajadhyax S., Kannan S., Kane S., Pagare S., Ranganathan K. (2014). Clinicopathological features and prognostic implications of loss of K5 and gain of K1, K8 and K18 in oral potentially malignant lesions and squamous cell carcinomas: Immunohistochemical analysis. Tumor Biol..

[74] Garrel R., Dromard M., Costes V., Barbotte E., Comte F., Gardiner Q., Cartier C., Makeieff M., Crampette L., Guerrier B. (2006). The Diagnostic Accuracy of Reverse Transcription-PCR Quantification of Cytokeratin mRNA in the Detection of Sentinel Lymph Node Invasion in Oral and Oropharyngeal Squamous Cell Carcinoma: A Comparison with Immunohistochemistry. Clin. Cancer Res..

[75] Chisholm J.C., Houliston E. (1987). Cytokeratin filament assembly in the preimplantation mouse embryo. Development.

[76] Oshima R.G., Baribault H., Caulín C. (1996). Oncogenic regulation and function of keratins 8 and 18. Cancer Metastasis Rev..

[77] Bauman P.A., Dalton W.S., Anderson J.M., Cress A.E. (1994). Expression of cytokeratin confers multiple drug resistance. Proc. Natl. Acad. Sci. USA.

[78] Chu Y.W., Seftor E.A., Romer L.H., Hendrix M.J. (1996). Experimental coexpression of vimentin and keratin intermediate filaments in human melanoma cells augments motility. Am. J. Pathol..

[79] Hendrix M.J., Seftor E.A., Chu Y.W., Seftor R.E., Nagle R.B., McDaniel K.M., Leong S.P., Yohem K.H., Leibovitz A.M., Meyskens F.L. (1992). Coexpression of vimentin and keratins by human melanoma tumor cells: Correlation with invasive and metastatic potential. J. Natl. Cancer Inst..

[80] Chu Y.W., Runyan R.B., Oshima R.G., Hendrix M.J. (1993). Expression of complete keratin filaments in mouse L cells augments cell migration and invasion. Proc. Natl. Acad. Sci. USA.

[81] Raul U., Sawant S., Dange P., Kalraiya R., Ingle A., Vaidya M. (2004). Implications of Cytokeratin 8/18 filament formation in stratified epithelial cells: Induction of transformed phenotype. Int. J. Cancer.

[82] Casanova M.L., Bravo del Moral A., Martinez-Palacio J., Fernández-Aceñero M.J., Villanueva C., Larcher F., Conti C., Jorcano J. (2004). Epidermal abnormalities and increased malignancy of skin tumors in human epidermal keratin 8-expressing transgenic mice. FASEB J. Off. Publ. Fed. Am. Soc. Exp. Biol..

[83] Bordeleau F., Galarneau L., Gilbert S., Loranger A., Marceau N. (2010). Keratin 8/18 modulation of protein kinase C-mediated integrin-dependent adhesion and migration of liver epithelial cells. Mol. Biol. Cell.

[84] Chaudhari P.R., Vaidya M.M. (2015). Versatile hemidesmosomal linker proteins: Structure and function. Histol. Histopathol..

[85] Geerts D., Fontao L., Nievers M.G., Schaapveld R.Q., Purkis P.E., Wheeler G.N., Lane E.B., Leigh I.M., Sonnenberg A. (1999). Binding of integrin alpha6beta4 to plectin prevents plectin association with F-actin but does not interfere with intermediate filament binding. J. Cell Biol..

[86] Dmello C., Sawant S., Alam H., Gangadaran P., Tiwari R., Dongre H., Rana N., Barve S., Costea D.E., Chaukar D. (2016). Vimentin-mediated regulation of cell motility through modulation of beta4 integrin protein levels in oral tumor derived cells. Int. J. Biochem. Cell Biol..

[87] Alam H., Kundu S.T., Dalal S.N., Vaidya M.M. (2011). Loss of keratins 8 and 18 leads to alterations in α6β4-integrin-mediated signalling and decreased neoplastic progression in an oral-tumour-derived cell line. J. Cell Sci..

